# Supplementary material for: Progression through mitosis promotes PARP inhibitor-induced cytotoxicity in homologous recombination-deficient cancer cells
Source: Nat Commun. 2017 Jul 17;8:15981. doi: 10.1038/ncomms15981 (PMC5520019; doi:10.1038/ncomms15981)
Supplement: Supplementary Information [file ncomms15981-s1.pdf]

File name: Supplementary Information
Description: Supplementary figures.

File name: Peer review file
Description:

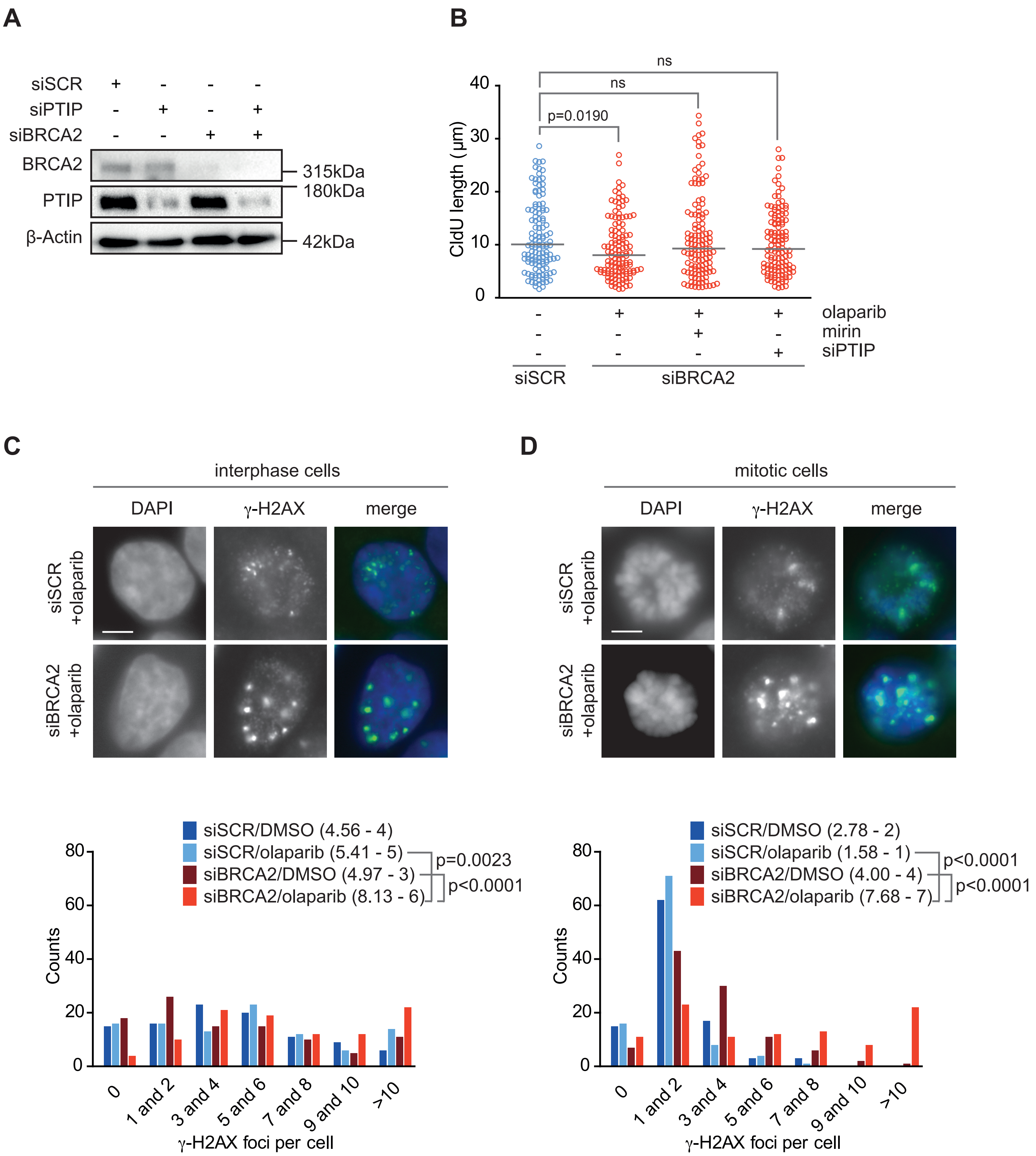

Supplementary Figure 1: PARP inhibition leads to MRE11/PTIP-dependent replication fork degradation in BRCA2 depleted cells, and ensuing mitotic DNA lesions. (A) Immunoblotting for BRCA2, PTIP and β-actin at 48 hours after transfection with indicated siRNAs in HeLa cells. **(B)** HeLa cells were transfected with indicated siRNAs and labeled with CldU. Cells were then treated with HU (5 mM) and DMSO, olaparib (0.5 μM) and/or mirin (50 μM) as indicated for 5 hours. DNA was spread into single fibers and CldU track length was determined of 125 fibers per condition. P values were calculated using two-tailed Mann-Whitney test. **(C, D)** HeLa cells were transfected with BRCA2 siRNA and treated with DMSO or olaparib (0.5 μM) for 24 hours. Cells were stained for γ-H2AX (green) and counterstained with DAPI (blue) and the number of γ-H2AX foci per nucleus were quantified for interphase cells (panel C) and mitotic cells (panel D). Scale bars indicate 5 μm. Per condition, 100 nuclei were analyzed. Indicated numbers between brackets represented averages and medians respectively. P values were calculated using a two-tailed Mann-Whitney test. Throughout the figure 'ns' indicates not significant.

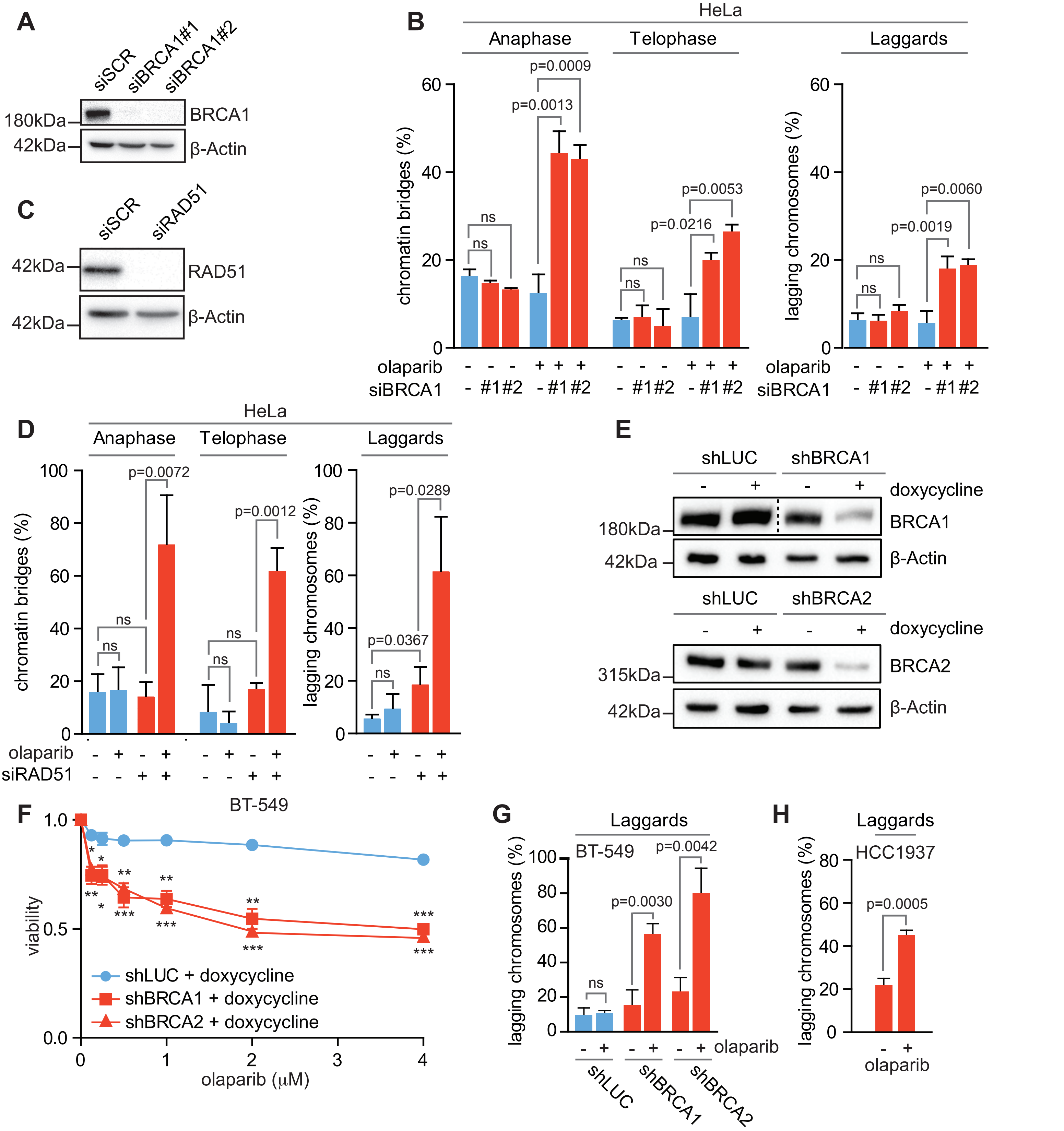

Supplementary Figure 2: PARP inhibition leads to chromatin bridges and lagging chromosomes in anaphase in HR-defective cancer cells. (A) Immunoblotting of BRCA1 and β-Actin at 48 hours after siRNA transfection in HeLa cells. **(B)** HeLa cells were transfected with indicated siRNAs and after 24 hours were treated with olaparib (0.5 μM) or DMSO for 24 hours. Percentages of cells containing chromatin bridges (n>20 events per condition per experiment) and lagging chromosomes (n>40 events per condition per experiment) were quantified. **(C)** Immunoblotting of RAD51 and β-Actin, at 48 hours after siRNA transfection in HeLa cells. **(D)** HeLa cells were transfected with indicated siRNAs and treated as for panel B. Percentages of cells containing chromatin bridges (n>20 events per condition) and lagging chromosomes (n>40 events per condition) were quantified. **(E)** Immunoblotting of BRCA1, BRCA2 and β-Actin at 4 days after doxycycline treatment of BT-549 cells, stably transduced with indicated doxycycline-inducible shRNAs. Dashed line indicates site where blot was cut. **(F)** BT-549 cells with indicated shRNAs were treated with doxycycline for 48 hours. Cells were subsequently treated with indicated olaparib concentrations for 4 days, after which MTT conversion was assessed. Averages and standard deviations of 4 independent experiments are shown. **(G)** BT-549 cells were pre-treated with doxycycline for 48 hours, and subsequently treated with olaparib (0.5 μM) or DMSO for 24 hours. Percentages of cells containing lagging chromosomes (n>40 events per condition) were quantified. **(H)** HCC1937 cells were treated with olaparib (0.5 μM) or DMSO for 24 hours. Percentages of cells containing lagging chromosomes (>40 events per condition) were quantified. Throughout the figure, P values were calculated using two-tailed Student's t-test. 'ns' indicates not significant, 'na' indicates not analyzable, * indicates p<0.05, ** indicates p<0.01, *** indicates p<0.001. All error bars indicate standard deviations of 3 independent experiments.

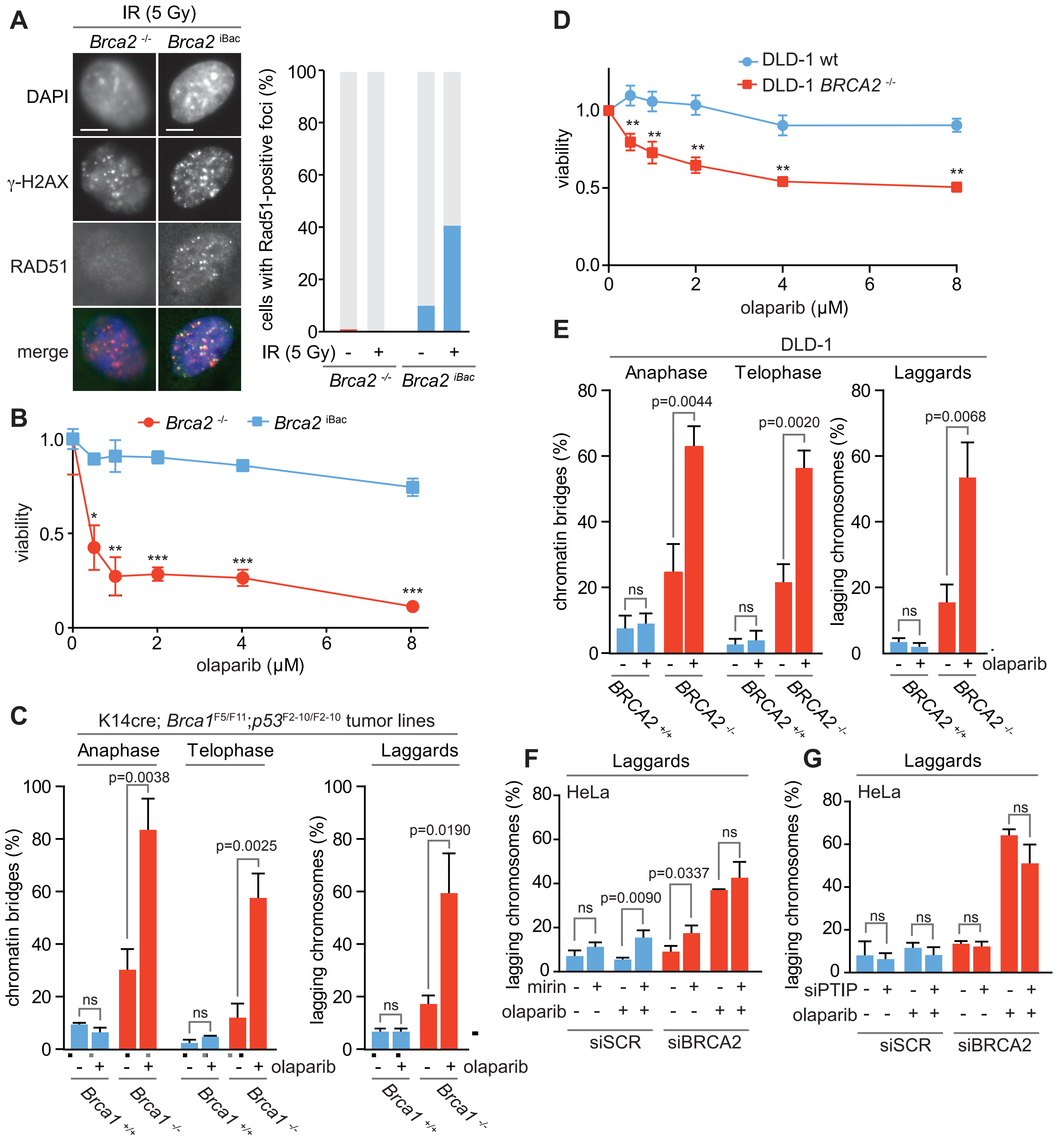

Supplementary Figure 3: PARP inhibition leads to chromatin bridges and lagging chromosomes in HR-defective cancer cells, and lagging chromosomes are not rescued by MRE11/PTIP inactivation. (A) KB2P1.21 (*Brca2*$^{-/-}$) and KB2P1.21R1 (*Brca2*^{iBac}) cells were irradiated (5 Gy), fixed after 6 hours and stained with for γ-H2AX (red) and RAD51 (green) and counterstained with DAPI (blue). Quantification of RAD51 foci is shown in the right panel. Nuclei with >5 RAD51 foci were considered positive and n>50 nuclei per condition were analyzed. Scale bars indicate 5 μm. **(B)** KB2P1.21 (*Brca2*$^{-/-}$) and KB2P1.21R1 (*Brca2*^{iBac}) cells were treated with indicated olaparib concentrations for 72 hours, after which viability was assessed by MTT conversion. Shown graphs represent averages from three replicates. **(C)** KB1P-B11 (*Brca1*$^{-/-}$) and KP3.33 (*Brca1*$^{+/+}$) cells were treated with olaparib (0.5 μM) or DMSO for 24 hours. Percentages of cells containing chromatin bridges (n>20 events per condition) and lagging chromosomes (n>40 events per condition) were quantified. **(D)** DLD-1 *BRCA2*$^{+/+}$ and *BRCA2*$^{-/-}$ cells were treated with indicated olaparib concentrations for 72 hours, after which viability was assessed by MTT conversion. Shown graphs represent averages from three replicates. **(E)** DLD-1 *BRCA2*$^{+/+}$ and *BRCA2*$^{-/-}$ cells were treated with olaparib (0.5 μM) or DMSO for 24 hours. Percentages of cells containing chromatin bridges (n>20 events per condition) and lagging chromosomes (n>40 events per condition) were quantified. **(F, G)** HeLa cells were transfected with indicated siRNAs and after 24 hours were treated with olaparib (0.5 μM) or DMSO and/or mirin (50 μM) for 24 hours. Percentages of cells containing lagging chromosomes (n>40 events per condition) were quantified. Throughout the figure, P values were calculated using two-tailed Student's t-test. 'ns' indicates not significant, * indicates p<0.05, ** indicates p<0.01, *** indicates p<0.001. All error bars indicate standard deviations of 3 independent experiments.

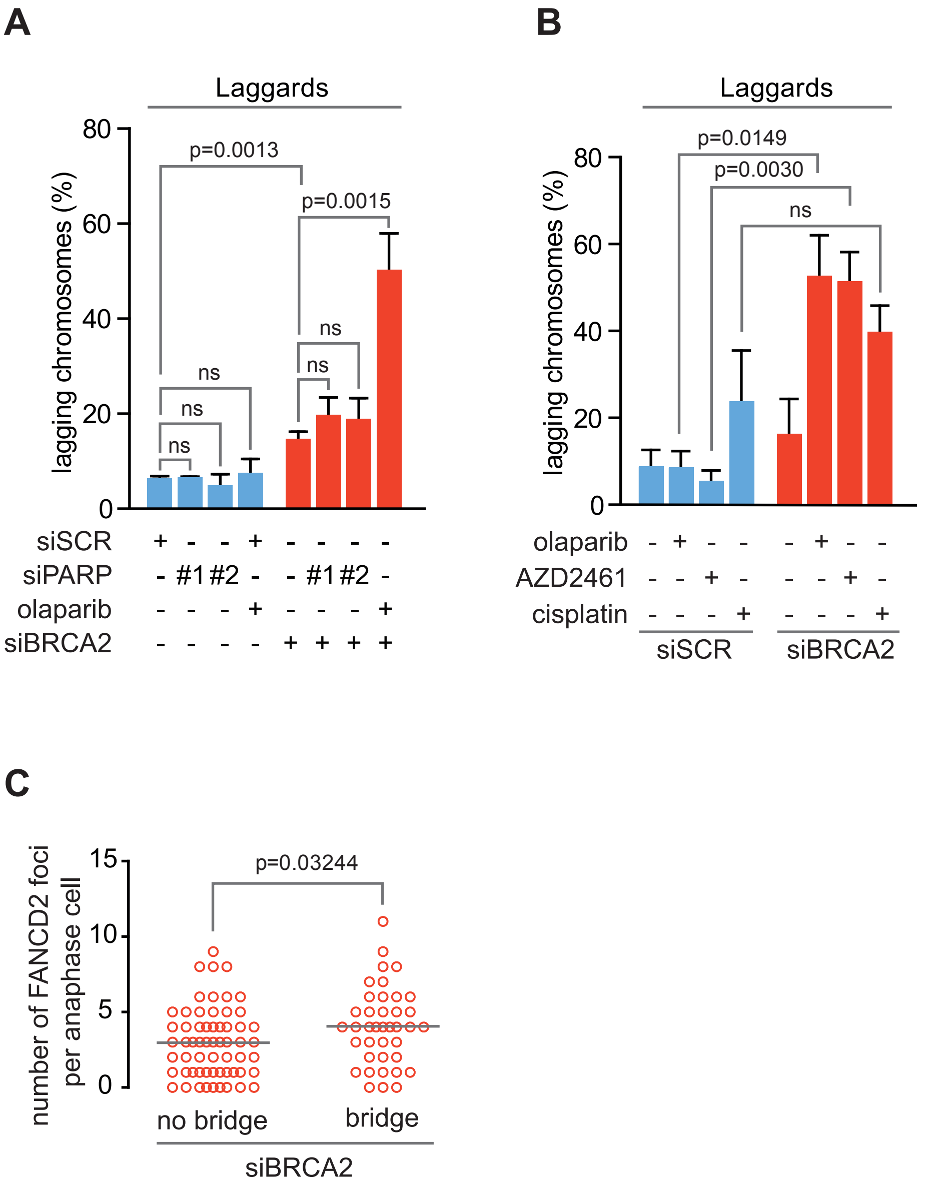

Supplementary Figure 4: PARP inhibition, but not PARP depletion, leads to lagging chromosomes in mitosis, and BRCA2-depleted cells with chromatin bridges have higher levels of mitotic DNA lesions. (A, B) HeLa cells were transfected with indicated siRNAs and after 24 hours were treated with olaparib (0.5 μM), AZD2461 (1 μM), cisplatin (1 μM) or DMSO for 24 hours. Percentages of cells containing lagging chromosomes (n>40 events per condition) were quantified. P values were calculated using two-tailed Student's t test. **(C)** HeLa cells were treated as for Figure 3E. BRCA2-depleted, olaparib-treated cells were harvested at 10 hours after release from thymidine, and anaphase cells were stained for FANCD2. Graph shows pooled data of cells treated with PARP inhibitor in S-phase and cells treated G2-phase. Foci were counted in 100 individual anaphase cells. For each cell, the presence of DAPI-positive chromatin bridges was determined. A Mann-Whitney test was performed to compare the number of foci in anaphase cells with and without chromatin bridges. Throughout the figure 'ns' indicates not significant. Horizontal bars indicate means, and all error bars indicate standard deviations of 3 independent experiments.

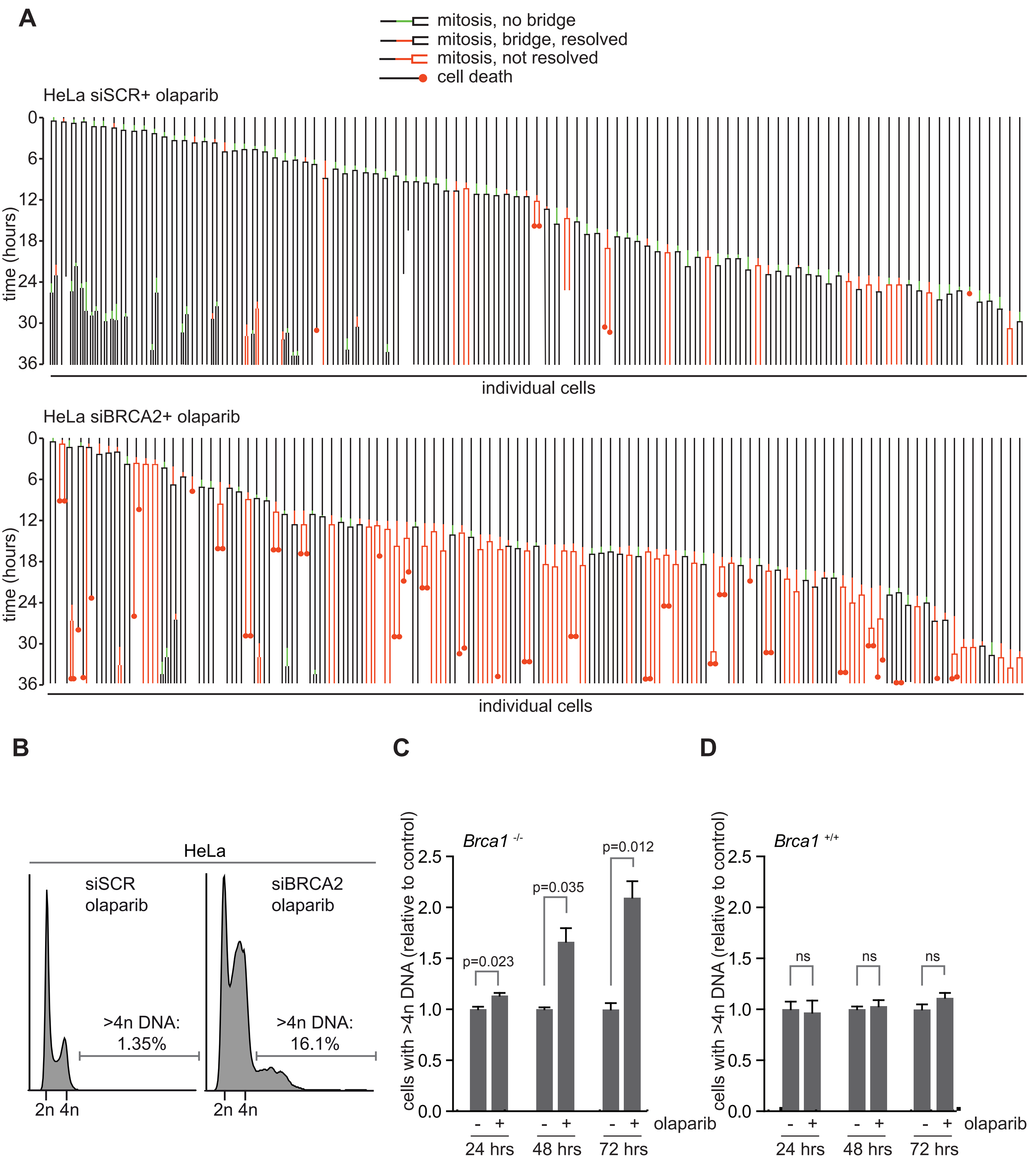

Supplementary Figure 5: (A) HeLa cells were transfected and treated as for Figure 4A. Cellular behavior of individual cells is plotted for control-transfected and siBRCA2-transfected HeLa cells treated with olaparib. **(B)** HeLa cells were transfected with indicated siRNAs for 24 hours and then treated with olaparib (0.5 μM) for 72 hours. Then, cells were fixed and DNA content was analyzed by flow cytometry. Indicated percentages show >4n DNA content. **(C, D)** KB1P-B11 cells (*Brca1*$^{-/-}$, panel C) and KP3.33 cells (*Brca1*$^{+/+}$, panel D) were treated with olaparib (0.5 μM) for 24, 48 or 72 hours after which cells were fixed and DNA content was analyzed. Percentages of cells with >4n DNA content are indicated. Error bars indicate standard deviations from three technical replicates. Throughout the figure 'ns' indicates not significant. P values were calculated using two-tailed Student's t test.

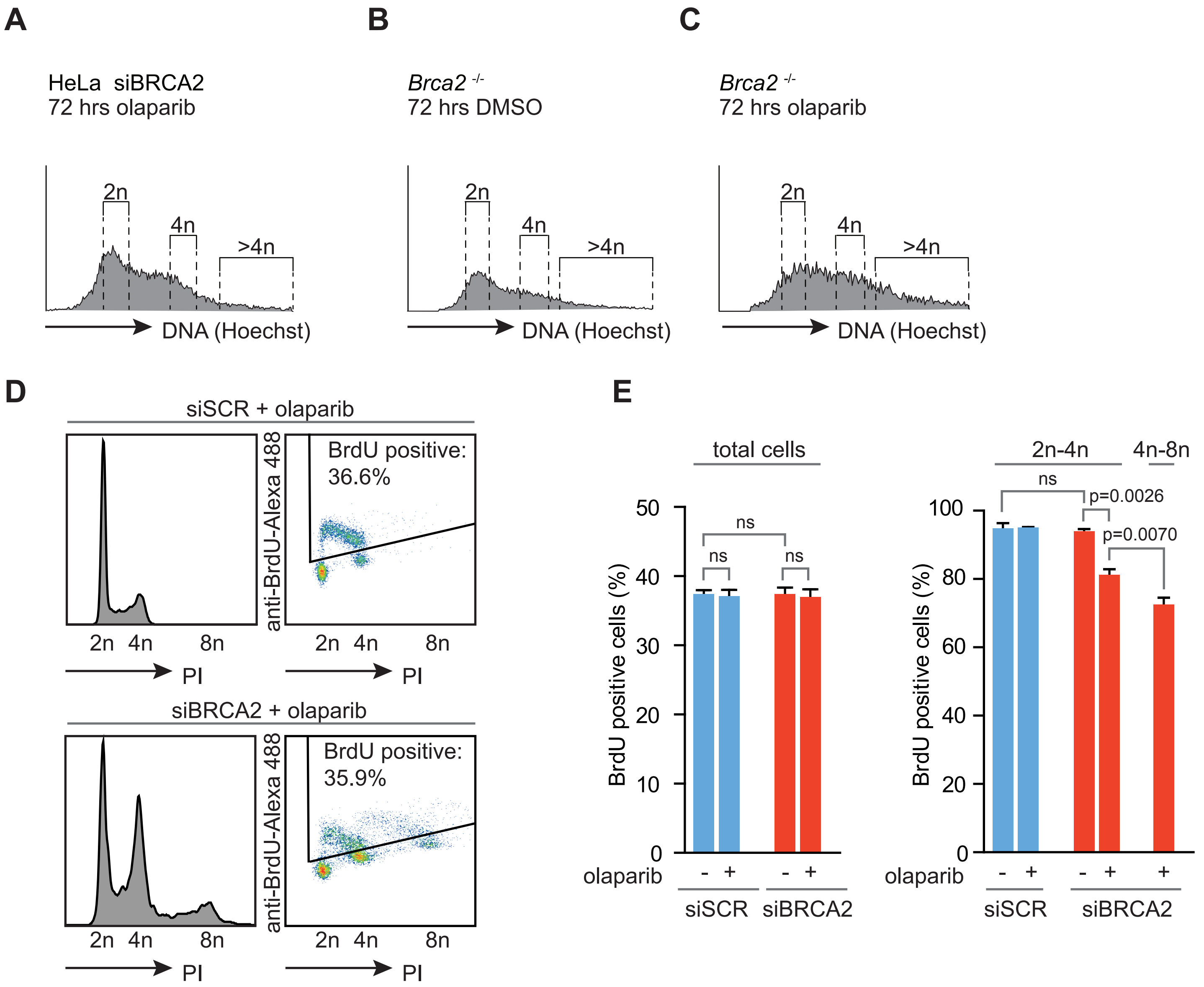

Supplementary Figure 6: PARP inhibition decreases, but does not block proliferation in BRCA2-defective cancer cells. (A) HeLa cells were transfected with siRNA targeting BRCA2 for 24 hours and were then treated with olaparib (0.5 μM) for 72 hours. Cells were then incubated with Hoechst for 45 min at 37°C, after which cells containing 2n, 4n or >4n were sorted as indicated. **(B, C)** KB2P1.21 (*Brca2*$^{-/-}$, panel B) and KB2P1.21R1 (*Brca2*^{iBac}, panel C) were treated, sorted and stained as described for panel A. **(D)** HeLa cells were transfected with indicated siRNAs and after 24 hours cells were treated with olaparib (1 μM) or DMSO for 72 hours. One hour prior to harvesting, cells were incubated with BrdU (10 μM). DNA content and BrdU-positivity was analyzed by flow cytometry. **(E)** Left panel: Quantification of BrdU-positive cells from panel D. Error bars indicate standard deviations of 3 independent experiments. Right panel: BrdU-positivity was determined for cells with DNA content between 2n and 4n, and for cells with DNA content between 4n and 8n. Error bars indicate standard deviations from three technical replicates. Throughout the figure 'ns' indicates not significant. P values were calculated using the Student's t-test.

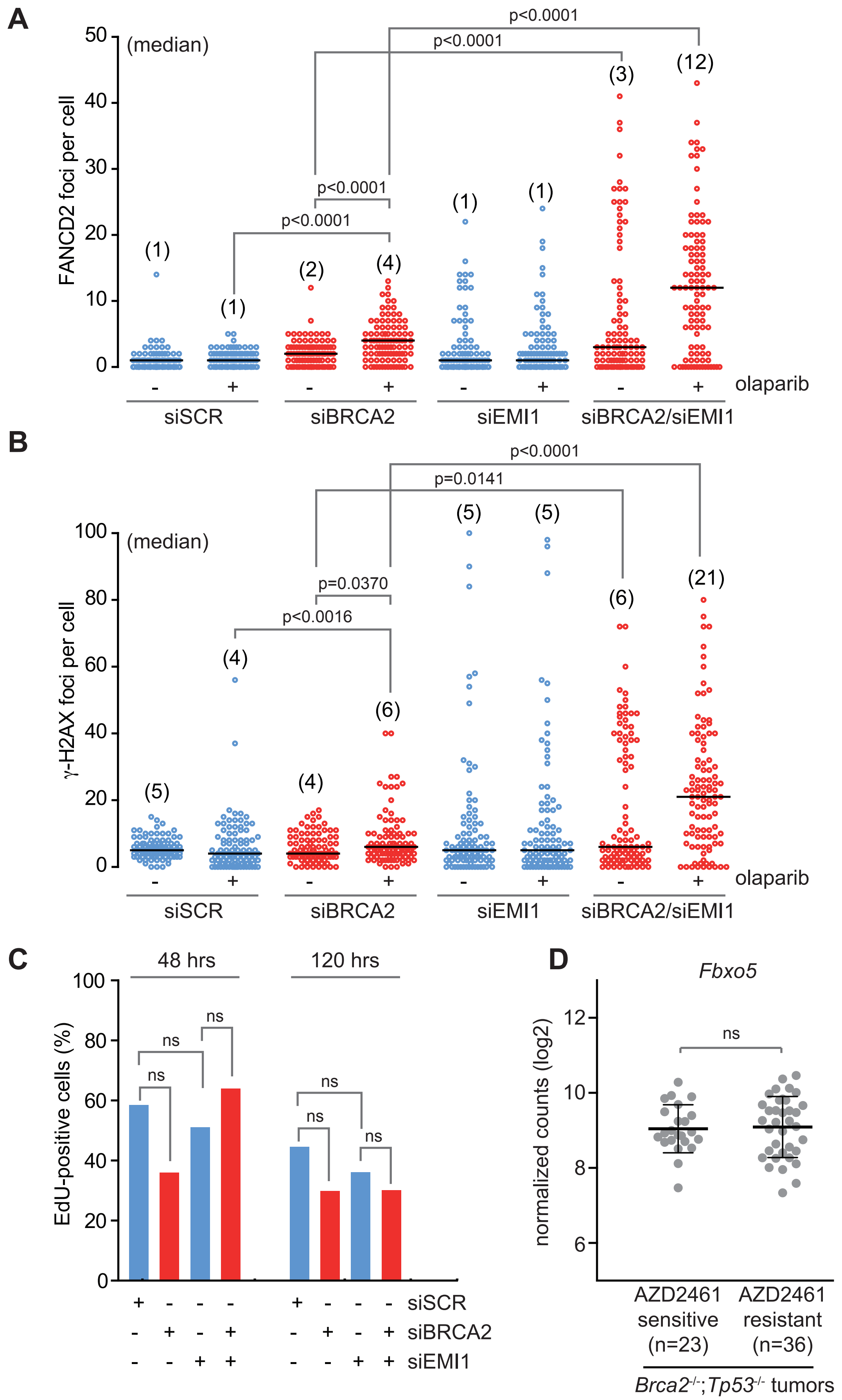

Supplementary Figure 7: EMI1 depletion does not rescue accumulation of DNA lesions nor cell proliferation in BRCA2-depleted cells, and is not differentially expressed in PARP inhibitor sensitive versus resistant tumors. Throughout the figure, control depleted cells are indicated with blue bars/dots, whereas BRCA2-depleted cells are indicated with red bars/dots **(A, B)** HeLa cells were transfected with indicated siRNAs for 24 hours and were then treated with DMSO or olaparib (0.5 μM) for 24 hours. Cells were stained for FANCD2 or and counterstained with DAPI, and the number of FANCD2 foci per nucleus (Panel A) or γ-H2AX foci per nucleus (panel B) were quantified in interphase cells. Per condition n=100 nuclei were analyzed. P values were calculated using two-tailed Mann-Whitney test. **(C)** HeLa cells were transfected with indicated siRNAs. After 48 hours, cells were incubated with EdU (10 μM) for 15 minutes and were subsequently fixed in 4% formaldehyde. EdU was conjugated to azide-Alexa488 and analyzed by fluorescence microscopy. At least 50 cells were analyzed per condition were analyzed. P values were calculated using two-tailed Student's t-test. **(D)** AZD2461 sensitive (n=23) or resistant (n=36) *Brca2*$^{-/-}$;*p53*$^{-/-}$ tumors were analyzed by RNA sequencing. Normalized counts for *Fbxo5* (encoding EMI1) are indicated. P value was calculated using two-tailed Student's t-test. Throughout the figure 'ns' indicates not significant.

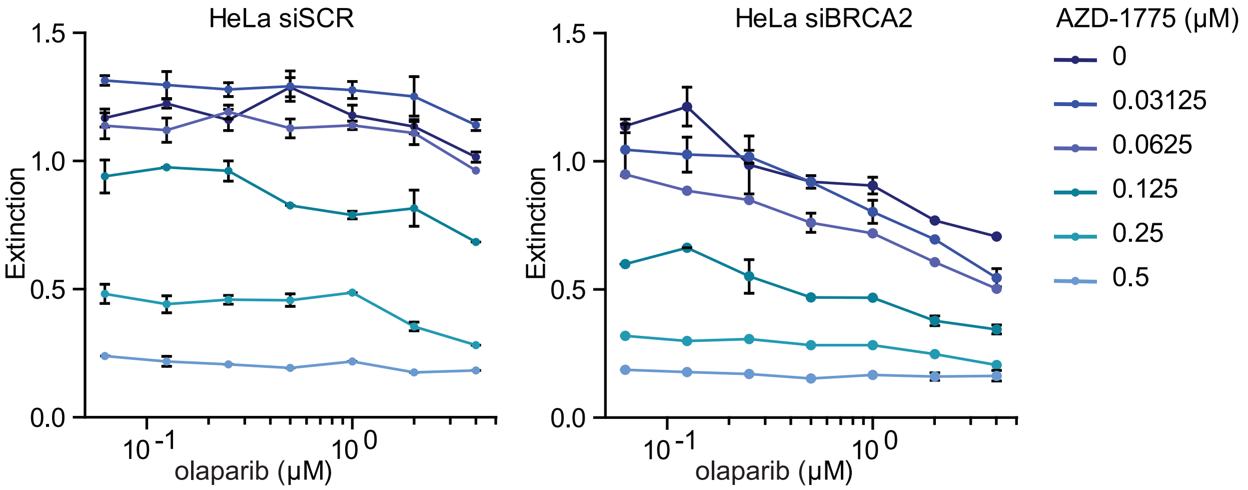

Supplementary Figure 8: Combined treatment of BRCA2-depleted HeLa cells with AZD-1775 and olaparib. HeLa cells were transfected with BRCA2 siRNA or control siRNA (SCR) for 24 hours and subsequently treated with indicated concentrations of olaparib and WEE1 inhibitor (AZD-1775) for 72 hours. MTT conversion was measured as a proxy for cell viability. Error bars indicate standard deviations of two independent experiments.

Supplementary Figure 9 part 1

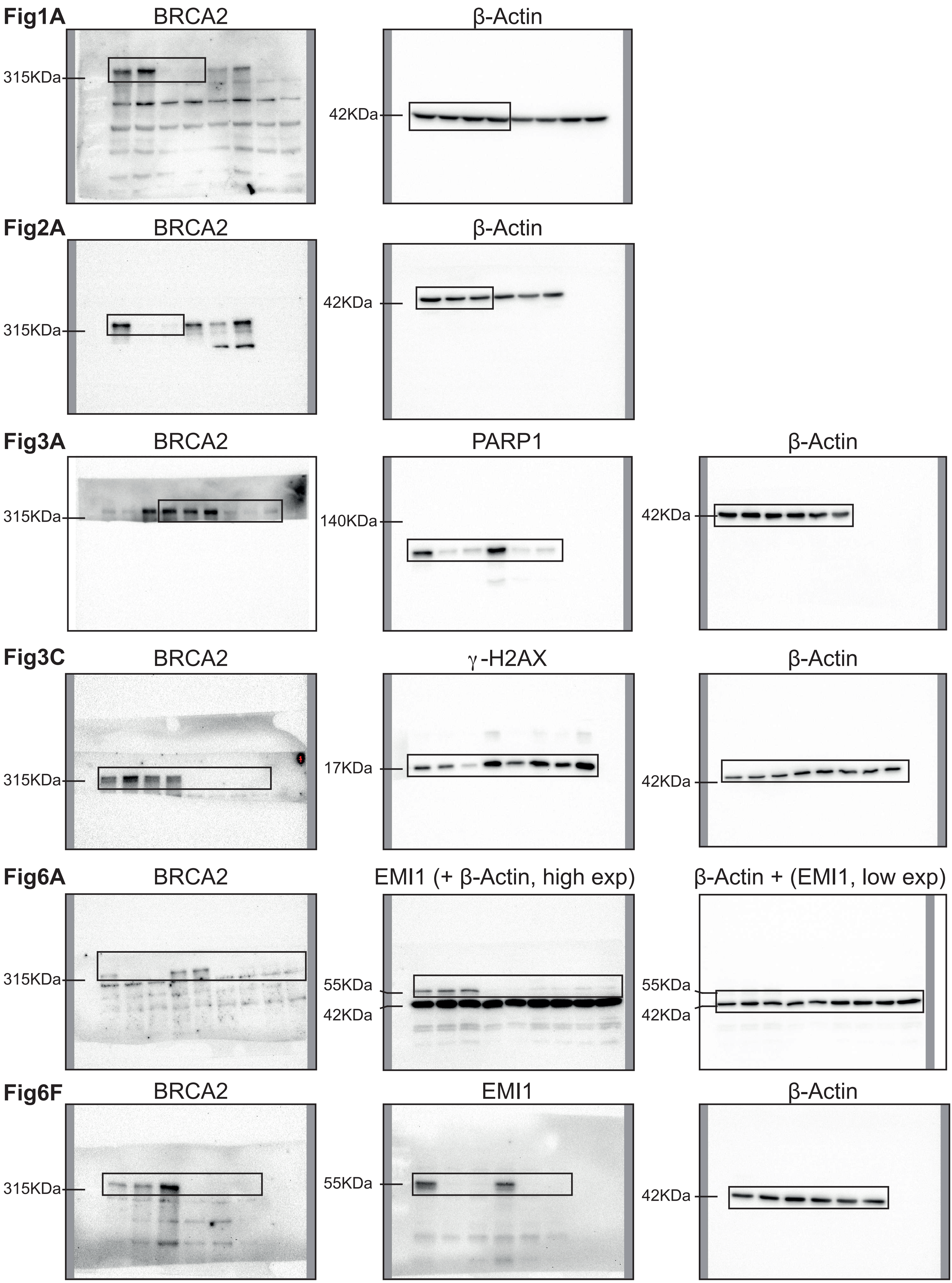

Supplementary Figure 9 part 2

Supplementary fig. 1A

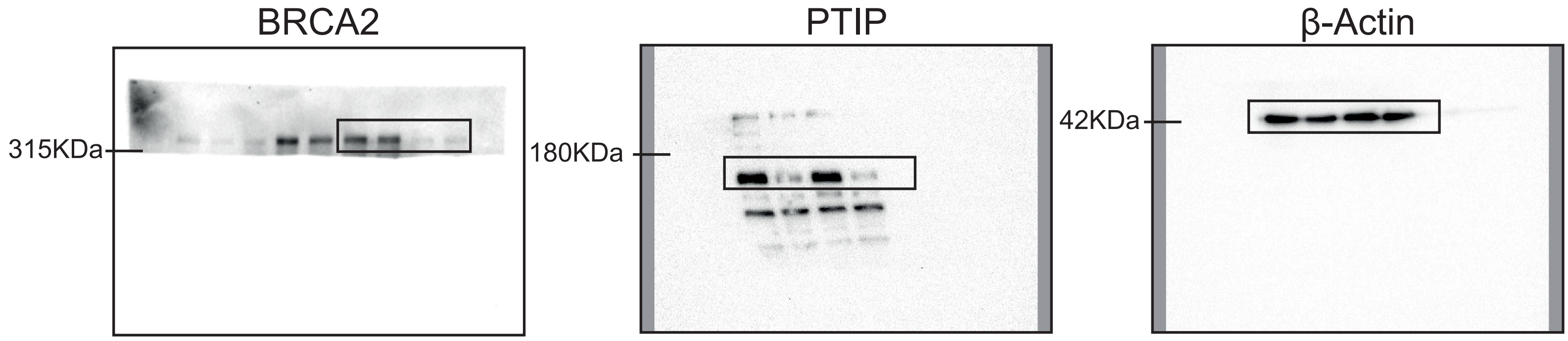

Supplementary fig. 2A

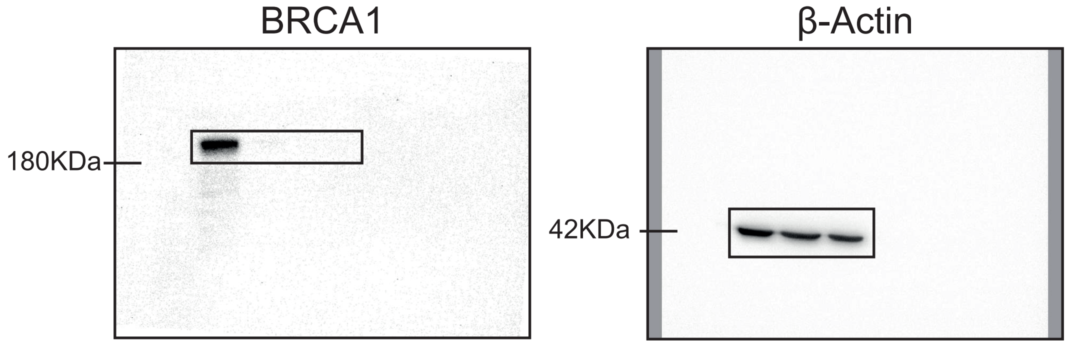

Supplementary fig. 2C

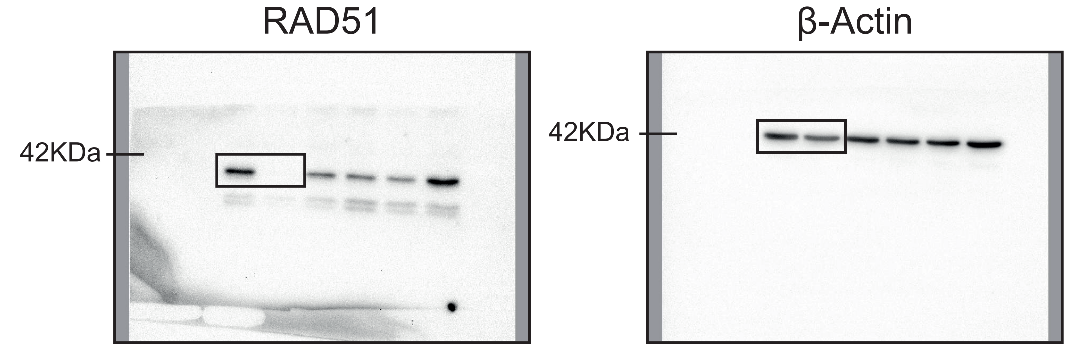

Supplementary fig. 2E

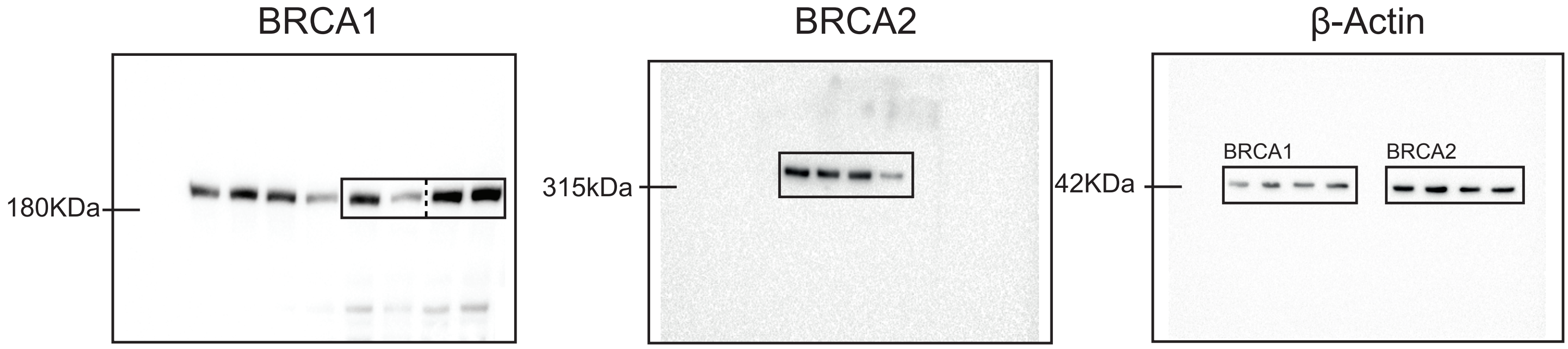

Supplementary Figure 9: Uncropped western blots.
